# Antibiotic susceptibility testing of *Burkholderia cepacia* complex isolates: an evidence-based guide for clinics

**DOI:** 10.3389/fmicb.2026.1779777

**Published:** 2026-02-26

**Authors:** Ekadashi Rajni, Indu Singh, Divakar Sharma, Kanika Bairwa, Megh Singh Dhakad

**Affiliations:** 1 Department of Microbiology, Mahatma Gandhi Medical College and Hospital, Jaipur, India; 2 School of Pharmacy, Graphic Era Hill University, Dehradun, India; 3 Department of Biotechnology, Graphic Era (Deemed to be) University, Dehradun, India; 4 Department of Microbiology, Maulana Azad Medical College and Associated Hospital, New Delhi, India

**Keywords:** antibiotic susceptibility testing (AST), *Burkholderia cepacia* complex (Bcc), clinical breakpoints, CLSI and EUCAST guidelines, opportunistic infections

## Introduction

The *Burkholderia cepacia* complex (Bcc) is a cluster of Gram-negative bacteria (non-fermenters) responsible for causing several opportunistic infections and outbreaks. This group of opportunistic bacteria is associated with several manifestations, including bloodstream infections, sepsis, pneumonia, surgical site infections, and conjunctival infections (Häfliger et al., 2020). Although conventionally associated with cystic fibrosis (CF) and chronic granulomatous disease, their epidemiology is evolving rapidly, as they are now encountered in other settings too, especially in immunocompromised hosts. In CF, certain Bcc species like *B. cenocepacia* drive “cepacia syndrome,” characterized by rapidly progressive pneumonia and mortality rates up to 5-fold higher than with other non-fermenters (Lipuma, 2010).

The clinical landscape often associated with Bcc infection comprises of prolonged hospital stay, previous antimicrobial exposure, and underlying immune dysfunctions. Several healthcare-associated outbreaks have also been linked to contaminated disinfectants, intravenous fluids, and medical devices (Courtwright, 2024; Ramos et al., 2019; Stone et al., in press). This epidemiological transition from a hitherto unknown pathogen to one known for its notoriety has underscored the importance of early identification and accurate antimicrobial susceptibility testing (AST), which are critical for effective management (Humphries et al., 2018). In addition to several intrinsic resistance determinants, such as reduced outer membrane permeability, chromosomally encoded β-lactamases, and overexpression of efflux pumps, Bcc also has the ability to develop adaptive or acquired resistance (Scoffone et al., 2017). Which observed during prolonged antimicrobial exposure, particularly in CF patients. Biofilm formation and target modification produce a milieu of complex dose–response relationships and poorly defined MIC endpoints, which further complicates AST.

Despite the growing clinical significance of Bcc, there are lacunae regarding an in-depth understanding of their AST (Wootton et al., 2021; Jorth et al., 2025). Firstly, there is poor agreement of AST among the broth microdilution, agar dilution, and disc diffusion methods used to determine accurate and reproducible minimum inhibitory concentrations (MICs). Secondly, poor correlation has been observed between reported MICs and clinical outcomes. Other difficulties include insufficient pharmacokinetic/pharmacodynamic correlation, high inter-laboratory variability, and paucity of data on clinical outcomes.

In keeping with the spirit of these facts, the Clinical and Laboratory Standards Institute has removed the Bcc breakpoints for disc diffusion from M100 ED34:2024 and has now eliminated MIC breakpoints as well (Clinical Laboratory Standards Institute, 2025). Instead, it refers to epidemiological cut-off values (ECVs) for the five primary drugs, namely ceftazidime, levofloxacin, meropenem, minocycline, and trimethoprim-sulfamethoxazole. These ECVs are used mainly for epidemiological purposes only. How useful these are going to be for clinicians in individual patient management is yet to be seen. This development also corroborates the European Committee on Antimicrobial Susceptibility Testing (EUCAST) guideline, which “does not recommend routine AST to guide therapy for Bcc.” It has also been documented in several cases that ECVs are found to be above MICs typically achievable by routine antibiotic dosing (European Committee on Antimicrobial Susceptibility Testing, 2013).

This current scenario is difficult to decipher for healthcare providers, as they are now increasingly encountering these organisms in clinical practice. The withdrawal of validated breakpoints has introduced uncertainty into the management of Bcc infections. Clinicians are frequently required to select therapy in the absence of reliable interpretive criteria, increasing the risk of inappropriate selection of antimicrobial treatment (Veeraraghavan and Walia, 2025).

Mukhida et al. stated that the “90-60 rule” in antimicrobial susceptibility testing of the infections caused by susceptible isolates, which responds to therapy in approximately 90% of cases, whereas those involving resistant strains still achieve success in about 60%, irrespective of specific pathogens, drugs, or infection sites (Mukhida et al., 2025). Regarding the Bcc, this 90-60 rule holds particular promise; stewardship emphasizing cotrimoxazole (historically >80% susceptibility) could avert overuse of carbapenems or cephalosporins, where susceptibility often fluctuates (Horsley et al., 2020; Rhodes and Schweizer, 2016). The other areas that need further exploration include the deployment of antibiotic combinations and the feasibility of developing infection-site–specific breakpoints. Progress in this field will depend on coordinated multicentre research linking *in vitro* susceptibility data with clinical outcomes, refinement of methodological standards, and renewed efforts toward evidence-based guideline development.

In summary, no standard, accurate method for Bcc AST has been described to date (except for broth microdilution using frozen panels). In light of recent developments, it is prudent to report only MICs with no breakpoints or interpretation (susceptible; S, intermediate; I, and resistant; R). While reporting ECVs may be considered, they are riddled with the inherent pitfall of being used as MICs by the clinicians. Not only are ECVs not species specific, but they are also much higher than the archived breakpoints (Clinical Laboratory Standards Institute, 2025). If reported, this could lead to clinical Bcc isolates being erroneously misinterpreted as being falsely susceptible. It is also critical to include a disclaimer admitting the lack of clinical breakpoints and that results need to be interpreted in the context of the patient's clinical condition.

## Discussion

These facts summarize the conundrum faced in reporting and interpreting the antibiotic susceptibility data for this group of intrinsically resistant non-fermenting Gram-negative bacilli (Veeraraghavan and Walia, 2025). Given their growing clinical significance, it is important that both laboratorians and clinicians are made aware of these significant loopholes. As part of surveillance, labs can document MIC data along with ECVs to contribute to evidence-based, meaningful patient care. This significant shift from traditional interpretive (S/I/R) to MIC-based reporting shall foster enhanced interaction between the diagnostic and therapeutic arms of the healthcare system.

## References

[B1] Clinical and Laboratory Standards Institute (2025). Performance Standards for Antimicrobial Susceptibility Testing, 35th Edn. CLSI Supplement M100. Wayne, PA: CLSI.

[B2] Courtwright A. (2024). Should antimicrobial resistance limit access to an organ transplant? AMA J. Ethics. 26, E367–372. doi: 10.1001/amajethics.2024.367 38700520

[B3] European Committee on Antimicrobial Susceptibility Testing (2013). Guidance Document: Antimicrobial Susceptibility Testing of Burkholderia cepacia Complex. Växjö: EUCAST.

[B4] Häfliger E. Atkinson A. Marschall J. (2020). Systematic review of healthcare-associated Burkholderia cepacia complex outbreaks: presentation, causes and outbreak control. Infect. Prev. Pract. 2:100082. doi: 10.1016/j.infpip.2020.100082 34368718 PMC8335909

[B5] Horsley A. Fothergill J. L. Walshaw M. J. (2020). Antibiotic treatment for *Burkholderia cepacia* complex in people with cystic fibrosis. Cochrane Database Syst. Rev. 2020:CD009529. doi: 10.1002/14651858.CD009529.pub4 PMC7117566 32239690

[B6] Humphries R. M. Ambler J. Mitchell S. L. Castanheira M. Dingle T. Hindler J. A. . (2018). Best practices for evaluation of antimicrobial susceptibility tests. J. Clin. Microbiol. 56:e01934-17. doi: 10.1128/JCM.01934-17 29367292 PMC5869819

[B7] Jorth P. Manuel C. McLemore T. Humphries R. M. Cole N. C. Schuetz A. N. . (2025). Evaluation of antimicrobial susceptibility testing methods for *Burkholderia cepacia* complex isolates. J. Clin. Microbiol. 63:e01480-24. doi: 10.1128/jcm.01480-24 39840992 PMC11837569

[B8] Lipuma J. J. (2010). The changing microbial epidemiology in cystic fibrosis. Clin. Microbiol. Rev. 23, 299–323. doi: 10.1128/CMR.00068-09 20375354 PMC2863368

[B9] Mukhida S. Das N. K. Kannuri S. Athavale P. Khan S. (2025). Does 90-60 rule help in reduction of anti-microbial resistance? If yes, then how? Fut. Microbiol. 20, 1163–1165. doi: 10.1080/17460913.2025.2598190 41355233 PMC12931686

[B10] Ramos K. J. Smith P. J. McKone E. F. Pilewski J. M. Lucy A. Hempstead S. E. . (2019). Lung transplant referral for individuals with cystic fibrosis: Cystic Fibrosis Foundation consensus guidelines. J. Cyst. Fibros. 18, 321–333. doi: 10.1016/j.jcf.2019.03.002 30926322 PMC6545264

[B11] Rhodes K. A. Schweizer H. P. (2016). Antibiotic resistance in *Burkholderia* species. Drug Resist. Updat. 28, 82–90. doi: 10.1016/j.drup.2016.07.003 27620956 PMC5022785

[B12] Scoffone V. C. Chiarelli L. R. Trespidi G. Mentasti M. Riccardi G. Buroni S. (2017). Burkholderia cenocepacia infections in cystic fibrosis patients: drug resistance and therapeutic approaches. Front. Microbiol. 8:1592. doi: 10.3389/fmicb.2017.01592 28878751 PMC5572248

[B13] Stone A. Nolt D. Ali R. Kim Y. Caverly L. J. Gold J. A. (in press). Investigation of a pseudo-outbreak of Burkholderia cepacia complex caused by contaminated phosphate-buffered saline. Am. J. Infect. Control. doi: 10.1016/j.ajic.2026.01.001. 41520924

[B14] Veeraraghavan B. Walia K. (2025). Breakpoint withdrawals and emerging evidence. Indian J. Med. Microbiol. 43:100905. doi: 10.1016/j.ijmmb.2025.100905 40582507

[B15] Wootton M. Davies L. Pitman K. Howe R. A. (2021). Evaluation of susceptibility testing methods for *Burkholderia cepacia* complex. Clin. Microbiol. Infect. 27, 788.e1–788.e4. doi: 10.1016/j.cmi.2020.11.012 33253940

